# Development of new genomic microsatellite markers from robusta coffee (*Coffea canephora *Pierre ex A. Froehner) showing broad cross-species transferability and utility in genetic studies

**DOI:** 10.1186/1471-2229-8-51

**Published:** 2008-04-30

**Authors:** Prasad Suresh Hendre, Regur Phanindranath, V Annapurna, Albert Lalremruata, Ramesh K Aggarwal

**Affiliations:** 1 Centre for Cellular and Molecular Biology (CCMB), Uppal Road, Tarnaka, Hyderabad- 500 007, Andhra Pradesh, India

## Abstract

**Background:**

Species-specific microsatellite markers are desirable for genetic studies and to harness the potential of MAS-based breeding for genetic improvement. Limited availability of such markers for coffee, one of the most important beverage tree crops, warrants newer efforts to develop additional microsatellite markers that can be effectively deployed in genetic analysis and coffee improvement programs. The present study aimed to develop new coffee-specific SSR markers and validate their utility in analysis of genetic diversity, individualization, linkage mapping, and transferability for use in other related taxa.

**Results:**

A small-insert partial genomic library of *Coffea canephora*, was probed for various SSR motifs following conventional approach of Southern hybridisation. Characterization of repeat positive clones revealed a very high abundance of DNRs (1/15 Kb) over TNRs (1/406 kb). The relative frequencies of different DNRs were found as AT >> AG > AC, whereas among TNRs, AGC was the most abundant repeat. The SSR positive sequences were used to design 58 primer pairs of which 44 pairs could be validated as single locus markers using a panel of arabica and robusta genotypes. The analysis revealed an average of 3.3 and 3.78 alleles and 0.49 and 0.62 PIC per marker for the tested arabicas and robustas, respectively. It also revealed a high cumulative PI over all the markers using both sib-based (10^-6 ^and 10^-12 ^for arabicas and robustas respectively) and unbiased corrected estimates (10^-20 ^and 10^-43 ^for arabicas and robustas respectively). The markers were tested for Hardy-Weinberg equilibrium, linkage dis-equilibrium, and were successfully used to ascertain generic diversity/affinities in the tested germplasm (cultivated as well as species). Nine markers could be mapped on robusta linkage map. Importantly, the markers showed ~92% transferability across related species/genera of coffee.

**Conclusion:**

The conventional approach of genomic library was successfully employed although with low efficiency to develop a set of 44 new genomic microsatellite markers of coffee. The characterization/validation of new markers demonstrated them to be highly informative, and useful for genetic studies namely, genetic diversity in coffee germplasm, individualization/bar-coding for germplasm protection, linkage mapping, taxonomic studies, and use as conserved orthologous sets across secondary genepool of coffee. Further, the relative frequency and distribution of different SSR motifs in coffee genome indicated coffee genome to be relatively poor in microsatellites compared to other plant species.

## Background

Coffee tree, a member of the family Rubiaceae, belongs to the genus *Coffea *that comprises > 100 species. Of these two species, the tetraploid *Coffea arabica *L. (i.e. arabica coffee; 2n = 4x = 44) and the diploid *C. canephora *Pierre ex A. Froehner (i.e. robusta coffee; 2n = 2x = 22), are cultivated commercially. Coffee, one of the most popular non-alcoholic beverages, is consumed regularly by 40% of the world population mostly in the developed world [1], and thus occupies a strategic position in the world socio-economy.

Efforts undertaken globally to improve coffee, though successful, have proven to be too slow and severely constrained owing to various factors. The latter includes: genetic and physiological makeup (low genetic diversity and ploidy barrier in arabicas, and self incompatibility/easy cross-species fertilization in robustas), long generation cycle, requirement of huge land resources, and equally the dearth of easily accessible and assayable genetic tools/techniques for screening/selection. The situation warrants recourse to newer, easy, practical technologies that can provide acceleration, reliability and directionality to the breeding efforts, and allow characterization of cultivated/secondary genepool for proper utilization of the available germplasm in genetic improvement programs. In this context, development of DNA marker tools and availability of markers-based molecular linkage maps becomes imperative for MAS-based accelerated breeding of improved coffee genotypes.

Among the different types of DNA markers, the Short Sequence Repeats (SSR) based microsatellite markers promise to be the most ideal ones due to their multi-allelic nature, high polymorphism content, locus specificity, reproducibility, inter-lab transferability and ease for automation [2]. Microsatellite markers have been developed for a large number of plant species and are increasingly being used for ascertaining germplasm diversity, linkage analysis and molecular breeding [3]. Despite these advantages, only ~180 microsatellite markers have been reported till to date for coffee [4-12], signifying the need for expanding the repertoire of these genetically highly informative markers for efficient management and improvement of coffee germplasm resources. Here we report, a set of 44 novel microsatellite markers developed by radioactive screening of a small-insert partial genomic library of *C. canephora *(robusta coffee). Interestingly, all these markers exhibit broad cross-species transferability. We also demonstrate their utility as genetic markers for ascertaining the germplasm diversity, genotype individualization, linkage mapping and taxonomic affinities.

## Results

The present study aimed to isolate new coffee-specific informative SSRs useful as genetic markers for characterizing coffee genome and linkage mapping studies. For the purpose, a partial small-insert genomic library was constructed from a commercially cultivated robusta variety 'Sln-274'. The library was screened using radioactive SSR oligo probes to isolate SSR-containing DNA fragments, which were sequenced and used for designing primer pairs from the flanking regions and subsequent conversion to PCR-based SSR markers. The designed primer pairs were standardized for PCR amplification, and then validated for utility as genetic markers using panels of elite coffee genotypes, a mapping population for linkage studies, and related taxa of coffee for cross-species transferability. In addition, sequence data of the screened and putative SSR-positive selected clones were used to assess the relative abundance of different SSR motifs in robusta coffee genome. In total 44 new highly informative SSR markers are developed.

### Screening/Identification of SSR positive genomic sequences from the small insert partial genomic library of Sln-274

The small-insert partial genomic library constructed from robusta variety Sln-274 comprised 15,744 clones. Radioactive screening of the arrayed and blotted clones indicated 446 putative positives of which good quality sequence data could be obtained for 199 clones. The average insert size of the sequenced clones was 773.5 bp. Considering the latter, and that the sequenced clones represented a random sample of the genomic library with respect to the size, the total size of the cloned genome amounted to 12.2 Mb which equaled to ca. 1.5 % of the robusta coffee genome [13] (Table 1). SSR search of the clone sequences using the MISA search module, detected 76 genuine SSR-positive clones (0.48% of the total library) containing both targeted and non-targeted SSR motifs. Overall, these clones contained 92 SSRs comprising DNRs (48.3%), TNRs (25.9%), and HO-NRs (4.8%), and 24 SSRs comprising only MNRs (20.7%) (Table 1, 2). Among the targeted repeat motifs (screened SSR-oligo nucleotides), AG was the most abundant repeat (26.7%), followed by AC (12.9%) and AGC (7.8%), whereas CCG (0.9%) was the least abundant and ACT was not detected at all (Table 2). Similarly, among the non-targeted SSR motifs other than MNRs, AT was the most abundant repeat (8.6%, Table 2).

**Table 1:** Summary statistics of screening of the small-insert partial genomic library of robusta coffee for putative SSR positive clones/sequences and SSRs.

***Summary of Screening/sequencing***	
Total Number of clones screened (X)	15,744
Number of clones selected and sequenced after screening	446 (2.83% of X)
Number of good quality sequences obtained	199 (1.27% of X)
Total number of SSR containing clones (Y)	76 (0.48% of X)
Number of sequences containing more than 1 SSR core	26 (34.21% of Y)
Number of sequences containing compound SSRs	15 (12.93% of Y)
Number of SSR+ sequences used for primer design/synthesis	58 (0.37% of X)
Number of working primer pairs	53 (0.34% of X)
Average size of the cloned/sequenced insert	773.5 bp
Haploid genome size of *C. canephora *[13]	809 Mb
Estimated genome screened (number of library clones x. average insert size)	12.2 Mb (1.5 % genome equivalent)
*C. canephora *genome sequenced (good quality sequences × average insert size)	0.15 Mb (0.01 % of robusta genome)

***Summary of SSRs identified in the library***	

Number of non-targeted MNRs of minimum 12-mer length (a)	24 (0.15% of X)
Number of targeted DNRs having a minimum of 6 repeats (b)	46 (0.29% of X)
Number of non-targeted DNRs having a minimum of 6 repeats (c)	10 (0.06% of X)
Total number of DNRs (b+c)	56 (0.36% of X)
Number of targeted TNRs having a minimum of 5 repeats (d)	30 (0.19% of X)
Total number of DNRs and TNRs (b+c+d)	86 (0.55% of X)
Total Number of non-targeted HO-NRs having a minimum of 5 repeats (e)	6 (0.04% of X)
Total Number of DNRs, TNRs and HO-NRs (b+c+d+e)	92 (0.58% of X)
Total Number of SSRs (a+b+c+d+e)	116 (0.73% of X)

**Table 2:** Summary statistics of distribution and abundance of detected SSRs in the tested genomic library and SSR frequency estimates for robusta coffee genome

**SSR motif**	SSRs detected in the library (% of total SSRs)	Mean no. of repeats/SSR (Range of repeat iterations in the SSR core)	Estimated number/distance of SSRs in the robusta coffee genome		
			
			Total SSRs/genome (X = *n.a/b*)*	SSRs/Mb genome (Y = X/a)	SSR spacing in the genome^@ ^(Z = 1000/Y)
*Targeted SSRs *(**DNRs^T ^**+ **TNRs^T^**)					

AG	31(26.7)	10.0 (6 to 29)	2057	2.5	393
AC	15 (12.9)	8.4 (6 to 14)	995	1.2	812
**DNRs^T^**	**46 (39.7)**	**9.6 (6 to 29)**	**3053**	**3.8**	**265**
AGC	9 (7.8)	6.8 (5 to 10)	597	0.7	1354
ATC	4 (3.5)	5.0 (5)	265	0.3	3048
ACG	3 (2.6)	6.7 (5 to 9)	199	0.3	4063
ACC	3 (2.6)	5.7 (5 to 7)	199	0.3	4063
AAT	3 (2.6)	5.3 (5 to 6)	199	0.3	4063
AAC	3 (2.6)	5.0 (5)	199	0.3	4063
AGG	2 (1.7)	6.0 (5 to 7)	133	0.7	6095
AAG	2 (1.7)	5.5 (5 to 6)	133	0.7	6095
CCG	1 (0.9)	6.0 (6)	66	0.1	12190
ACT	0	--	--	--	--
**TNRs^T^**	**30 (25.9)**	**5.9 (5 to 10)**	**1991**	**2.5**	**406**
**SSRs^T^**	**76 (65.5)**	**8.3 (5 to 29)**	**5044**	**6.2**	**160**

*Non-targeted DNRs *(**DNRs^NT^)**					

AT/AT	10 (8.6)	10.3 (6 to 23)	50563^#^	62.50	16

*Miscellaneous non-targted SSRs*					

A/T	21 (18.1)	nc			
C/G	3 (2.6)				
Note: Three of these MNRs were detected as part of the compound SSR motifs					
AAAT	2 (1.7)	Nc			
AAGTGG	2 (1.7)				
AATT	1 (0.7)				
AAAAAT	1 (0.7)				
**DNRs^T+NT^**	**56 (48.3)**	**11.5 (6 to 29)**	**53616**	**66.3**	**15**
**DNRs^T+NT ^& TNRs^T^**	**86 (74.1)**	**9.5 (5 to 29)**	**55607**	**68.7**	**15**

nc: Not calculated

*: X = estimated number of SSRs in genome; n = No. of detected SSRs in the library; a = 809 Mb -size of the haploid robusta genome [13]; b = 12.19 Mb- size of the screened robusta genome (see table 1)

^#^: b = 0.16 Mb -size of genome sequenced

^@^: Distance (in Kb) between two consecutive SSRs

^T^: Targeted SSRs; ^NT^: Non-targeted SSRs

### Frequency and distribution of SSRs in coffee genome

A total of 76 targeted SSRs (DNRs and TNRs) and 10 non-targeted DNRs were assessed for their lengths, distribution in the present library, and their relative abundance in the robusta genome (Table 2). Average length (in terms of repeat units) for the DNRs and TNRs was 9.6 and 5.9, respectively. Among DNRs, AT and AG were comparable and longer than AC, whereas ACG and AGC were the longest of the TNRs (Table 2). The size of cloned/screened genomic library and the observed data for identified SSRs were considered along with the earlier predicted size of the robusta genome [13] to derive relative estimates for frequency/distribution of different SSR motifs in the robusta genome. The analysis revealed coffee genome to be enriched in AT type DNRs (AT-DNR), which were estimated to be many fold more than any other SSR motifs (targeted and/or non-targeted). The results indicated one AT-DNR per 16 Kb (1/16 Kb) of robusta genome; this was almost 20-fold higher than the next most abundant DNR *i.e*. AG (ca. 1/393 Kb). The DNRs as a single class were estimated to be 1/15 Kb genome when AT (comprising 94% of the total DNRs) was included, and 1/265 Kb coffee genome for the remaining ones. In comparison, the overall frequency of TNRs was calculated to be 1/406 Kb with AGC being the most predominant (ca. 1/1300 Kb) and CCG the least (ca. 1/12200 Kb). In addition, a few other higher order SSRs (mainly the AT-rich) were also detected but these were not used for estimate calculations, as their numbers were very low. Thus, the present study indicated an abundance of one SSR (either DNR or TNR) per 15 Kb of robusta coffee genome, wherein the DNRs were ~27 times more abundant than the TNRs.

### Development of microsatellite markers

All the identified SSR-positive sequences were tried to design primer pairs for conversion to microsat markers using 'SSR motif length' (of ≥ 7 and 5 repeats for DNRs and higher order SSRs, respectively) as one major criterion. As a result, only 56 of the total 92 identified SSRs (all except MNRs) were found suitable for primer design indicating 60.9% primer suitability. These comprised 42.2% DNRs, 40.7% compound SSRs, 6.8% TNRs, 5.1% TtNRs and 1.7% HNRs. In addition, primers were also designed for 2 of the randomly chosen 14 MNRs to test their potential for conversion to SSR markers. Among the SSRs found unsuitable for primer design, 70.6% had shorter motif length and 29.4% had flanking regions unsuitable for primer modeling. Of the 58 potential primer pairs designed, 52 could be successfully amplified and 44 of these could further be validated (Table 3, 4) as useful markers indicating ~76% primer to marker conversion ratio.

**Table 3:** Details of the newly developed SSR primers

Sl. No.	Primer Id	Primer sequence (F: Forward; R: reverse)	Repeat unit	Ta (°C)	Amplicon (bp)	GenBank accession No.	Linkage group
1	CaM02	F: CGCCAGCCACAGCCACTTGC	(AGG)7	50	224	EU526557	--
		R: GCGGGGGTAAGAAAGAGGCGAG					
2	CaM03	F: CGCGCTTGCTCCCTCTGTCTCT	(AC)11	57	173	EU526558	CLG03
		R: TGGGGGAGGGGCGGTGTT					
3	CaM06	F: ACCCGATATTCAACCGACATGC	(CT)7	50	278	EU526559	--
		R: CATGACTTGAGCGCTAATATTTGAT					
4	CaM08	F: CAGCTGAAGTGGTGAAAAACAAGAG	(TC)8	50	202	EU526560	
		R: CGCTTTCTTGTTTTCTCCATTTCAG					--
5	CaM09	F: CAGGAAGAGAAGAAAGTGAAATTGAC	(TC)8	50	137	EU526560	
		R: CGCTTTCTTGTTTTCTCCATTTC					
6	CaM11	F: GTCCCCGCTTAAATAATATACACACA	(AC)8–15 bp-AC(6)(AT)6	50	285	EU526561	--
		R: ATAGGACGGAGGGAGTAATAGAATAAA					
7	CaM12	F: TTCGGGCTCACCTGGCAG	(CAG)10	50	155	EU526562	
		R: CGCGGAAGCAGGACATGGATT					--
8	CaM13	F: CCTCGCCCTCAATCACCTCCTAG	(AAAT)5	50	287	EU526563	
		R: GGCTCCCCAAGAATCCTCAACTC					--
9	CaM15	F: AGCCCTAGACGAGATGGATTCC	(CAG)5	50	170	EU526564	
		R: CGGCTCCTTCTGCACTCCCATTT					
10	CaM16	F: AAGGCAGCTGAAGCGGGACAAA	(TC)11	50	199	EU526565	CLG11
		R: TGGGGAGAGCTGCAGTTGGAGG					
11	CaM17	F: CGGGCGTTTCTTCTTTTGAGTTGC	(GTC)6	50	212	EU526566	--
		R: TCACGGTTTCTCAAGTCGGGGATTTA					
12	CaM18	F: CCGACTTGGACTGATGCGAAATTGA	(TC)9	57	181	EU526567	--
		R: AAAGCAAAAAACCAGAAAACACGAAGA					
13	CaM20	F: GAAACCGCTGAAATTCGGTA	(TATGGG)3	57	217	EU526568	CLG16
		R: CCCTCTGATTTCTCCTTTCATC					
14	CaM21	F: GGGCTTACCGACCGCTCACAG	(TC)8	57	161	EU526569	--
		R: CCGCTATTGTTGCTGCTATGGAGTTG					
15	CaM22	F: CCCCTCCTCCTCCTACTAGATGGTGGT	(AT)15	57	113	EU526570	CLG02
		R: GGTCCAGGGTCCATCCATTCTTGA					
16	CaM23	F: TGCTTGTAAGGGAATTTCTGGTCAG	(AATT)5	50	154	EU526571	--
		R: GTGCGAATGTGGAACCTTTTAAGTCA					
17	CaM24	F: GGATTCGACAAGGTTGGCAGAGC	(CCT)5–87 bp-(CTG)6	57	193	EU526572	--
		R: TGCCGAAGAAGAGGGAGATAGTGATG					
18	CaM25	F: TCCATCTTCCTTCATTTCTGCTGCTAA	(GA)9	57	186	EU526573	--
		R: CCTTCACCCCCTTTGCACTTCCTTA					
19	CaM26	F: CGTTGCCATTTCTTCCCTTCTTTCTTC	(TG)7–21 bp-(GA)9	57	236	EU526574	--
		R: ACACCTTACCCCCTTATCGTTTAGAA					
20	CaM27	F: AAGAGTGTTTGGGATTGCATTTTTAT	(TA)7(GT)14	55	178	EU526575	--
		R: CCGCGTAGGCTTTGTTTGG					
21	CaM30	F: TTGCCTTCCGGATTTTTGATTCA	(CA)6(TA)5	50	222	EU526576	--
		R: AGTTCTAAGGCTGAGGCGGCTAAAG					
22	CaM31	F: ATCCACTGCTGTCACCTTTTGTTA	(TAA)5	55	261	EU526577	--
		R: AGCAGTGTGTGTGTTAAAGAGGAGTT					
23	CaM32	F: CAGACAGACCAGAGAGAGACACCTAAC	(TA)12	50	204	EU526577	CLG12
		R: CCCCCTCCAAAATAATTCAGAAAA					
24	CaM33	F: GCGCATTAGGCGTGGGAGAA	(A)13–5 bp-(AG)18	55	240	EU526578	--
		R: CAGAGGTTGTCGGTCAGGTGGAGAA					
25	CaM34	F: CTCCAAATTATTAAGCACAACAAACAA	(GA)10	55	202	EU526579	--
		R: ATCCGCCTCCAGGTCTTATCC					
26	CaM35	F: CGAGCTAGAATGGATGACTTGGTTGG	(TGGAAG)5	55	203	EU526580	CLG04
		R: GTTGCTCGCACCCGCTTCC					
27	CaM36	F: TGGTTTTAGTTTGTTTATTTTGATGTGAT	(TTA)7	55	185	EU526581	--
		R: CGAGCCCTCCCCTTGCA					
28	CaM38	F: GAAGCTGAAGCGGGAGGGTAGTAATT	(G)13(GA)7	55	228	EU526582	--
		R: CCCATCCACCCAACCTTCATTTC					
29	CaM39	F: GAGCAGAGGGAGACGGTGTGGT	(GA)12	50	196	EU526583	--
		R: CGCGCAACTCTTCGAACTCTAACC					
30	CaM40	F: TTGACACGAAACAGGAAATAAATATAG	(CGA)8	55	238	EU526584	--
		R: CCCTTCCCCTCATAGCCCTTT					
31	CaM41	F: CATCGTCTCCATCGTTGCTCTATC	(TAAA)5	55	242	EU526585	--
		R: CCCTCCCCCTCTTTCCTATCTAAT					
32	CaM42	F: TGGGTCAAGGATCCGTGTAAGAAAGA	(CT)8	55	191	EU526586	CLG01
		R: CCCTCACCAGTTCCCGATGTCAG					
33	CaM43	F: CCTGACCGTGAACCTGACCGTGAC	(CT)8	55	202	EU526587	--
		R: TCGGGACTTGTTTTGGTTTTTGGGT					
34	CaM44	F: TGCTCTTGCCCTCTTTCATCC		55	222	EU526588	CLG09
		R: TCCCGAAAAAGAAAATAAGATAAAGAG	(CT)9				
35	CaM45	F: CGCGGCCAGTGAATTCGAGCTC	(GT)8(GA)5	50	218	EU526589	--
		R: TCGCCATTTGGAGCTGCTGATTCA					
36	CaM46	F: TGGTGCGGTGTTTTTCAGTTTGGAGA	(AT)9 (AC)12	55	222	EU526590	CLG11
		R: AACCACCCACGCCCACCAATTAAAT					
37	CaM49	F: CCGGTTAATACATTGGTCTTT	(A)33	55	200	EU526591	--
		R: ATGACATTGTTGACTTTGCTATAA					
38	CaM52	F: TGCCACTCGGAGCTCACTTCA	(CCG)6	55	160	EU526592	--
		R: GGCTGCCGAGGTTCCAATT					
39	CaM53	F: TTAGGTGTGAGGAGGGATGGGACTG	(GGC)9	50	172	EU526593	--
		R: CCACAGACTCCTCGTTCGGCAATC					
40	CaM54	F: ACGGGTGAGTCGAAGGGGGAGCAGT	(GGCAGA)4–22 bp-(GCA)9	50	185	EU526593	--
		R: CACGCCGGCCCACATCTCGAAA					
41	CaM55	F: ATGGGGGGTGTCGGTCTATGTGA	(GA)4(G)4 (A)27	50	183	EU526594	--
		R: CGCAATTCGCTGTCACCTCCG					
42	CaM57	F: CGAACTCGAACTCAAGCTCAGA	(TA)23	50	190	EU526595	--
		R: AAGGATATATACGGTAATTTTA					
43	CaM58	F: ACCCCCTCTCCCTCTCCATTTTTAC	CAGA(CA)7	55	192	EU526596	--
		R: GCACGAGGATGGAGCAGAGCACT					
44	CaM59	F: AAGTGAGTGGTTGTGGCATTAAAT	GATA(GA)8	50	229	EU526591	--
		R: TTCTTACAAAATCTCATCCCCTCAT					

CaM: ***Ca***nephora ***M***icrosatellite marker; '--': Unmapped; these were not polymorphic among parents of the tested mapping population; CLG: Combined Linkage Group (as per [13]). The amplicon size is based on the original clone of Sln-274 genomic library from which the marker was designed.

**Table 4:** Allelic diversity attributes of new SSR markers as revealed across elite genotypes of arabica and robusta, and related coffee taxa

Primer Id	*C. arabica *(n = 8)						*C. canephora *(n = 8)						*Coffea *spp. (n = 12)			*Psilanthus *spp. (n = 2)		
				
	N_A_	PA^$^	Allele range	H_o_	H_e_	PIC	N_A_	PA^$^	Allele range	H_o_	H_e_	PIC	N_A_	PA^$^	Allele range	N_A_	PA^$^	Allele range
CaM02	2	0	252–262	Duplicated loci			2	0	256–268	0.71	0.69	0.67	8	4^a,c,k,l^	252–278	2	0	262–272
CaM03	6	1^6^	164–184	0.38	0.74*	0.70	6	0	171–194	0.63	0.88	0.83	12	5^c,e,f,j,l^	165–201	1	1^m^	187
CaM06	3	0	285–327	Duplicated loci			2	0	275–277	1.00	0.53*	0.56	4	1^j^	275–289	2	0	275–281
CaM08	1	0	210	Monomorphic			3	1^16^	201–205	0.50	0.43	0.40	4	2^b,l^	142–201	1	1^n^	254
CaM09	1	0	135	Monomorphic			3	1^16^	135–139	0.50	0.43	0.40	7	5^a,b,g,l^	124–211	NA	--	--
CaM11	1	0	286	Monomorphic			1	0	286	Monomorphic			4	2^c,h^	278–295	NA	--	--
CaM12	1	0	137	Monomorphic			4	1^10^	122–137	1.00	0.65**	0.61	4	1^g^	124–137	2	0	131–137
CaM13	2	1^5^	281–286	0.00	0.23	0.23	1	0	286	Monomorphic			7	3^j,k^	278–336	2	1^n^	255–283
CaM15	2	0	167–170	Duplicated loci			1	0	167	Monomorphic			2	1^l^	164–167	2	2^m,n^	153–156
CaM16	3	0	187–198	0.63	0.51	0.49	4	1^11^	181–198	0.75	0.74	0.72	9	2^c,l^	177–198	2	0	191–193
CaM17	2	0	175–181	0.88	0.53	0.55	2	0	175–181	0.63	0.46	0.48	3	1^l^	162–181	2	0	175–181
CaM18	5	0	180–189	Duplicated loci			5	0	178–186	0.38	0.79**	0.75	12	5^d,e,j,k^	174–189	1	0	175
CaM20	2	0	184–192	0.13	0.46	0.48	3	0	192–200	0.13	0.42*	0.40	3	1^d^	192–198	NA	--	--
CaM21	2	0	158–164	Duplicated loci			4	1^10^	158–162	0.25	0.64**	0.62	9	3^a,j,l^	154–178	3	3^m,n^	161–172
CaM22	1	0	103	Monomorphic			6	2^9,16^	99–110	0.43	0.86**	0.80	8	2^c,l^	82–122	1	0	88
CaM23	1	0	154	Monomorphic			1	0	154	Monomorphic			3	1^l^	140 154	2	2^m,n^	152–158
CaM24	2	0	191–197	0.00	0.50**	0.52	4	0	191–198	0.43	0.71	0.67	8	4^a,b,g,l^	178–204	2	2^m,n^	177–189
CaM25	4	0	182–185	0.13	0.53**	0.5	3	0	182–184	0.63	0.51	0.48	5	0	182–186	2	0	182–186
CaM26	3	0	252–259	0.00	0.43**	0.42	5	0	247–255	0.25	0.80**	0.76	11	3^g,h,k^	241–262	1	0	254
CaM27	3	0	150–169	0.13	0.34	0.33	3	0	161–169	0.88	0.68	0.64	8	2^a,c^	150–169	2	0	161–168
CaM30	2	0	216–229	0.13	0.13	0.12	2	0	216–218	0.63	0.46	0.48	7	2^f,j^	212–229	4	3^m,n^	210–225
CaM31	3	0	258–261	Duplicated loci			4	0	258–262	0.38	0.59	0.57	6	2^h,k^	258–267	1	1^m^	265
CaM32	4	0	127–145	0.88	0.69	0.68	5	1^14^	145–158	0.75	0.72	0.68	10	2^a,e^	127–164	3	1^m^	133–145
CaM33	3	2^1,6^	230–233	0.13	0.34	0.32	7	2^12,13^	226–241	0.71	0.88	0.83	11	5^b,d,f,i,k^	213–143	1	1^m^	217
CaM34	2	0	194–199	Duplicated loci			2	0	198–200	0.00	0.23	0.23	5	2^e,l^	194–209	2	2^m,n^	166–171
CaM35	3	0	192–211	Duplicated loci			4	0	198–211	0.63	0.69	0.66	7	1^g^	186–211	1	0	204
CaM36	5	3^3,7,8^	228–253	0.00	0.85**	0.78	7	6^except 10,15^	230–268	0.17	0.92**	0.86	10	8 ^a,c,e,f,h,i,h,l^	181–262	1	1^n^	190
CaM38	6	14	214–226	0.38	0.86**	0.81	5	2^9,10^,^12,15^	227–235	0.17	0.80**	0.74	6	2^b,d^	220–241	2	0	223–227
CaM39	2	0	174–186	Duplicated loci			3	0	180–194	1.00	0.59*	0.60	9	2^b,h^	174–205	3	3^m,n^	208–229
CaM40	5	15	230–240	0.40	0.82	0.75	7	1^16^	226–242	0.50	0.91*	0.86	8	2^d,e^	232–246	3	0	233–239
CaM41	6	4^5,6,8^	232–243	0.25	0.68**	0.65	5	1^9^	234–242	0.25	0.81**	0.77	4	0	235–242	2	1^n^	237–244
CaM42	2	0	192–196	0.75	0.50	0.52	2	0	190–192	0.00	0.53*	0.56	7	2^e,l^	173–199	2	2^m,n^	191–195
CaM43	3	0	196–211	Duplicated loci			4	2^11,15^	198–203	0.63	0.64	0.64	10	4^b,c,e,f^	188–224	2	1^n^	192–196
CaM44	2	16	215–217	0.00	0.23	0.23	2	0	224–226	0.00	0.23	0.23	10	3^c,h,l^	194–227	3	1^n^	221–227
CaM45	3	0	151–182	0.50	0.43	0.42	5	2^10,13^	151–235	0.75	0.6	0.57	8	3^d,i,k^	147–214	3	1^m^	145–193
CaM46	4	2^3,6^	208–228	0.38	0.69	0.65	6	1^14^	208–223	0.38	0.82**	0.78	7	1^e^	208–234	2	1^n^	208–212
CaM49	3	0	191–194	0.38	0.68**	0.66	4	0	190–194	0.71	0.76	0.72	8	3^g,j,k^	186–194	NA	--	--
CaM52	2	0	157–159	0.00	0.23	0.23	3	0	148–158	0.13	0.34	0.33	5	0	148–158	1	0	155
CaM53	1	0	172	Monomorphic			3	2^2^	167–190	0.13	0.24	0.23	5	3^i,j,l^	125–197	2	2^m,n^	170–184
CaM54				No amplification			2	0	176–184	0.57	0.44	0.45	1	0	184	1	1^n^	164
CaM55	2	0	144–151	Duplicated loci			6	2^15,16^	159–178	0.75	0.84	0.79	9	0	144–178	1	0	160
CaM57	3	0	146–188	Duplicated loci			5	0	102–176	0.63	0.77	0.73	9	2^a,l^	102–174	3	1^m^	102–156
CaM58	2	0	189–191	Duplicated loci			2	0	183–191	0.75	0.5	0.52	8	2^b,l^	181–224	2	0	192–193
CaM59	2	0	224–226	0.13	0.13	0.12	3	0	222 –225	0.88	0.69	0.67	4	0	222–228	3	0	222–228

Range	0–6	0–4	--	0–0.88	0.13–0.86	0.12–0.81	1–7	0–6	--	0–1.00	0.23–0.88	0.23–0.83	1–13	0–8	--	0–4	0–3	--
Mean	2.7	0.37	--	0.29	0.5	0.49	3.78	0.67	--	0.52	0.63	0.62	7.07	2.42	--	1.98	0.87	--
SD (±)	1.4	0.87	--	0.28	0.22	0.21	1.73	1.13	--	0.29	0.19	0.18	2.87	1.72	--	0.79	0.95	--
SE (±)	0.3	0.13	--	0.06	0.05	0.04	0.26	0.17	--	0.04	0.03	0.03	0.43	0.26	--	0.12	0.14	--

^$^: Represents the genotype(s) as per Table 7, wherein the private allele is observed; *: Significant HW dis-equilibrium at P < 0.05; **: Highly significant HW dis-equilibrium at P < 0.01; Markers showing 100% H_o _values in arabicas, which are expected to be the result of duplicated loci were not considered for various estimates.

### Validation of microsatellite markers for use in genetic studies

#### Germplasm characterization

##### Allelic diversity, heterozygosity status and extent of polymorphism

**Figure 1:**
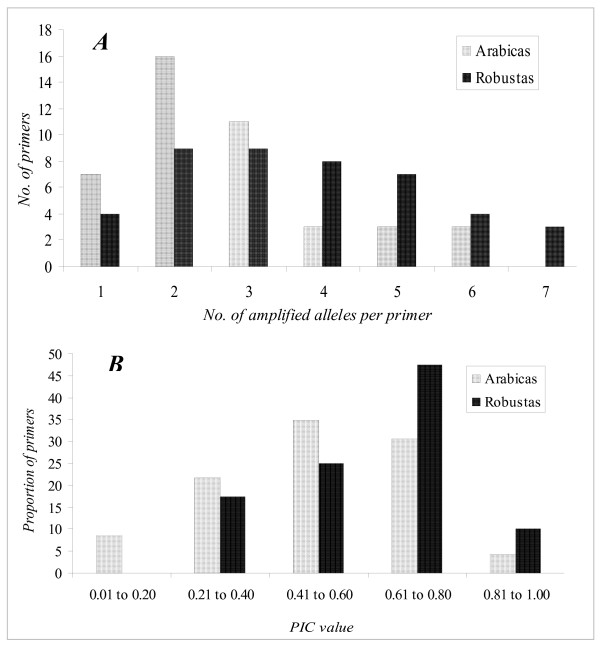
**Bar-graph showing comparative distribution of: (A) number of alleles (NA) amplified, and (B) PIC values of the new SSR markers in the tested sets of genotypes of arabica and robusta coffee.** Note: in case of PIC the plotted values represent normalized proportions of only the total polymorphic markers (which were 41 for robustas, 36 for arabicas, and only 23 in case of Arabica after removing the possible duplicate loci).

For ascertaining the useful attributes of genetic markers, all the new 44 microsatellite markers were tested on a panel of 16 elite robusta and arabica genotypes. Good allelic amplification was obtained for all the markers across the tested genotypes, except for CaM54 that did not give any amplification for the arabicas. In general, the new markers revealed low to medium allelic diversity, and notably 13 of them (CaM02, 06, 15, 18, 21, 31, 34, 35, 39, 43, 55, 57, 58) resulted in double alleles in case of all the tested arabicas. Overall, a maximum of six and seven alleles (N_A_) with an average of 2.7 and 3.8 alleles/marker were obtained for the tested markers of which 83.7% and 90.9% were polymorphic/informative forarabica and robusta genotypes respectively (Table 4). Seven markers (CaM08, 09, 11, 12, 22, 23, 53) in the case of arabicas and four (CaM11, 13, 15, 23) for robustas were found to be monomorphic. The distribution of number of alleles amplified by each polymorphic marker (P*m*) was highly skewed for arabica genotypes (Kurtosis: 1.19 and Skewness: 1.22) in comparison with robustas (Kurtosis: -1.08 and Skewness: -0.57) as seen in Figure 1a.

The PIC values varied considerably for the new markers across the tested genotypes. The mean PIC value for arabicas was 0.49 (range 0.12 – 0.81), which was significantly less than 0.62 (0.23 – 0.83) observed for robusta (Table 4, Figure 1b). Further, the student's t test revealed highly significant differences in the total number of amplified alleles (N_A_) and PIC value estimates for arabica and robusta genotypes (N_A_: t = 3.18, P = 0.00, and PIC: t = 3.46, P = 0.00) for the amplified and comparable markers.

The above SSR allelic data, when used to calculate the heterozygosity estimates, revealed highly significant differences between the observed and expected heterozygosity both for arabicas (mean H_o_: 0.29 and mean H_e _= 0.50; paired t value = 3.64; P = 0.00) as well as for robustas (mean H_o_: 0.52 mean H_e_: 0.63; paired t value = -2.54; P = 0.01). The results, thus, suggested significant heterozygote deficiency in both the germplasm sets. Further, only 15 of the 23 P*m*s (62.5%) were found to be in HW equilibrium in the case of arabicas, while the remaining eight showed significant heterozygote deficiency (Table 4) corroborating the heterozygosity data. Similarly, in robustas, 28 (65.2%) of the 41 P*m*s were found to be in HW equilibrium and of the remaining 14 P*m*s, eight markers showed significant heterozygote deficiency while six markers showed heterozygote excess.

The LD test performed for all the P*m*s, showed 29.8% (82 of 275) and 25.0% (202 of 780) pair-wise comparisons in significant dis-equilibrium (P < 0.05) for arabicas and robustas respectively. On an average each P*m *was found to be in dis-equilibrium with 3.4 (SD: ± 2.4, SE: ± 0.51) other P*m*s in case of arabicas and 4.9 (SD: ± 4.0, SE: ± 0.63) for robustas. The maximum LD was observed for the marker CaM24 (with six other markers) in arabicas and CaM26 (with eight other markers) in robustas.

##### Discriminatory power (individualization capacity) of novel SSR markers

The discriminatory power of all the new informative SSR markers for possible genotype individualization were inferred by calculating two types of the 'probability of identity' (PI) estimates i.e. sib-based and unbiased considering the tested germplasm as related or unrelated, respectively. PI estimates obtained (Table 5), show that the sib-based PI values for individual markers were around 10^-1 ^for both the arabicas and robustas, whereas the unbiased PI estimates ranged from 10^-1 ^– 10^-4 ^for arabicas and 10^-1 ^– 10^-3 ^for robustas. In comparison, the cumulative PIs indicating discriminatory power of the new markers were found to be manifold higher for the tested robusta genepool compared to arabicas. The sib-based cumulative PIs calculated over 10, 20 and total number of most informative markers (23 in the case of arabicas and 40 in the case of robustas) were: 4.28 × 10^-4^, 8.39 × 10^-6^, 5.29 × 10^-6 ^for arabicas, and 5.1 × 10^-5^, 1.81 × 10^-8^, 1.22 × 10^-12 ^for robustas. Similarly, comparable unbiased cumulative PI estimates were: 2.14 × 10^-15^, 4.59 × 10^-20^, 1.09 × 10^-20 ^for arabicas, and 2.68 × 10^-20^, 4.54 × 10^-32^, 2.05 × 10^-43 ^for robustas.

**Table 5:** Individual and cumulative probability of identity (PI) estimates calculated for the new polymorphic SSR markers for the tested elite arabica and robusta genotypes

*C. arabica*						*C. canephora*					
	
Sib-based estimates for PI			Unbiased estimates for PI			Sib-based estimates for PI			Unbiased estimates for PI		
			
*Marker*	*Individual*	*Cumulative*	*Marker*	*Individual*	*Cumulative*	*Marker*	*Individual*	*Cumulative*	*Marker*	*Individual*	*Cumulative*
CaM38	3.64 × 10^-1^	3.64 × 10^-1^	CaM03	9.67 × 10^-4^	9.67 × 10^-4^	CaM36	3.37 × 10^-1^	3.37 × 10^-1^	CaM40	2.47 × 10^-3^	2.47 × 10^-3^
CaM36	3.82 × 10^-1^	1.39 × 10^-1^	CaM41	5.80 × 10^-3^	5.61 × 10^-6^	CaM40	3.46 × 10^-1^	1.17 × 10^-1^	CaM36	3.12 × 10^-3^	7.69 × 10^-6^
CaM40	4.07 × 10^-1^	5.66 × 10^-2^	CaM38	1.36 × 10^-2^	7.65 × 10^-8^	CaM03	3.54 × 10^-1^	4.13 × 10^-2^	CaM33	3.15 × 10^-3^	2.42 × 10^-8^
CaM03	4.33 × 10^-1^	2.45 × 10^-2^	CaM36	1.36 × 10^-2^	1.70 × 10^-9^	CaM33	3.56 × 10^-1^	1.47 × 10^-2^	CaM03	9.15 × 10^-3^	2.22 × 10^-10^
CaM41	4.69 × 10^-1^	1.15 × 10^-2^	CaM40	1.36 × 10^-2^	4.88 × 10^-11^	CaM22	3.70 × 10^-1^	5.44 × 10^-3^	CaM22	1.58 × 10^-2^	3.50 × 10^-12^
CaM32	4.73 × 10^-1^	5.44 × 10^-3^	CaM25	1.14 × 10^-1^	5.55 × 10^-12^	CaM55	3.74 × 10^-1^	2.04 × 10^-3^	CaM55	1.64 × 10^-2^	5.75 × 10^-14^
CaM46	4.73 × 10^-1^	2.57 × 10^-3^	CaM32	1.20 × 10^-1^	6.64 × 10^-13^	CaM46	3.90 × 10^-1^	7.94 × 10^-4^	CaM46	2.25 × 10^-2^	1.29 × 10^-15^
CaM49	4.87 × 10^-1^	1.25 × 10^-3^	CaM46	1.20 × 10^-1^	7.94 × 10^-14^	CaM41	3.96 × 10^-1^	3.13 × 10^-4^	CaM38	2.38 × 10^-2^	3.08 × 10^-17^
CaM25	5.77 × 10^-1^	7.23 × 10^-4^	CaM49	1.56 × 10^-1^	1.24 × 10^-14^	CaM26	4.00 × 10^-1^	1.26 × 10^-4^	CaM26	2.86 × 10^-2^	8.79 × 10^-19^
CaM16	5.93 × 10^-1^	4.28 × 10^-4^	CaM16	1.73 × 10^-1^	2.14 × 10^-15^	CaM18	4.06 × 10^-1^	5.10 × 10^-5^	CaM57	3.05 × 10^-2^	2.68 × 10^-20^
CaM17	5.99 × 10^-1^	2.56 × 10^-4^	CaM26	2.14 × 10^-1^	4.59 × 10^-16^	CaM38	4.09 × 10^-1^	2.09 × 10^-5^	CaM18	3.74 × 10^-2^	1.00 × 10^-21^
CaM24	6.14 × 10^-1^	1.57 × 10^-4^	CaM45	2.49 × 10^-1^	1.14 × 10^-16^	CaM57	4.20 × 10^-1^	8.77 × 10^-6^	CaM41	3.95 × 10^-2^	3.97 × 10^-23^
CaM42	6.14 × 10^-1^	9.65 × 10^-5^	CaM27	3.13 × 10^-1^	3.58 × 10^-17^	CaM49	4.34 × 10^-1^	3.18 × 10^-6^	CaM32	4.21 × 10^-2^	1.67 × 10^-24^
CaM20	6.04 × 10^-1^	6.17 × 10^-5^	CaM33	3.13 × 10^-1^	1.12 × 10^-17^	CaM16	4.41 × 10^-1^	1.68 × 10^-6^	CaM24	6.02 × 10^-2^	1.01 × 10^-25^
CaM26	6.44 × 10^-1^	3.97 × 10^-5^	CaM20	3.49 × 10^-1^	3.92 × 10^-18^	CaM32	4.52 × 10^-1^	7.57 × 10^-7^	CaM45	6.92 × 10^-2^	6.96 × 10^-27^
CaM45	6.52 × 10^-1^	2.59 × 10^-5^	CaM24	3.57 × 10^-1^	1.40 × 10^-18^	CaM24	4.59 × 10^-1^	3.47 × 10^-7^	CaM49	8.67 × 10^-2^	6.03 × 10^-28^
CaM27	7.12 × 10^-1^	1.85 × 10^-5^	CaM42	3.57 × 10^-1^	5.00 × 10^-19^	CaM35	4.71 × 10^-1^	1.64 × 10^-7^	CaM35	8.74 × 10^-2^	5.27 × 10^-29^
CaM33	7.12 × 10^-1^	1.31 × 10^-5^	CaM17	3.67 × 10^-1^	1.84 × 10^-19^	CaM59	4.75 × 10^-1^	7.77 × 10^-8^	CaM16	9.19 × 10^-2^	4.84 × 10^-30^
CaM13	7.99 × 10^-1^	1.05 × 10^-5^	CaM13	5.00 × 10^-1^	9.18 × 10^-20^	CaM02	4.79 × 10^-1^	3.72 × 10^-8^	CaM31	9.46 × 10^-2^	4.58 × 10^-31^
CaM44	7.99 × 10^-1^	8.39 × 10^-6^	CaM44	5.00 × 10^-1^	4.59 × 10^-20^	CaM27	4.87 × 10^-1^	1.81 × 10^-8^	CaM21	9.90 × 10^-2^	4.54 × 10^-32^
CaM52	7.99 × 10^-1^	6.71 × 10^-6^	CaM52	5.00 × 10^-1^	2.30 × 10^-20^	CaM21	5.03 × 10^-1^	9.10 × 10^-9^	CaM59	1.41 × 10^-1^	6.40 × 10^-33^
CaM30	8.88 × 10^-1^	5.95 × 10^-6^	CaM30	6.89 × 10^-1^	1.58 × 10^-20^	CaM12	5.03 × 10^-1^	4.58 × 10^-9^	CaM02	1.48 × 10^-1^	9.46 × 10^-34^
CaM59	8.88 × 10^-1^	5.29 × 10^-6^	CaM59	6.89 × 10^-1^	1.09 × 10^-20^	CaM43	5.08 × 10^-1^	2.33 × 10^-9^	CaM27	1.56 × 10^-1^	1.47 × 10^-34^
CaM02	DL					CaM45	5.26 × 10^-1^	1.22 × 10^-9^	CaM43	1.63 × 10^-1^	2.40 × 10^-35^
CaM06	DL					CaM31	5.33 × 10^-1^	6.53 × 10^-10^	CaM12	1.68 × 10^-1^	4.05 × 10^-36^
CaM15	DL					CaM39	5.47 × 10^-1^	3.57 × 10^-10^	CaM25	1.73 × 10^-1^	7.02 × 10^-37^
CaM18	DL					CaM25	5.93 × 10^-1^	2.12 × 10^-10^	CaM08	2.49 × 10^-1^	1.75 × 10^-37^
CaM21	DL					CaM06	5.94 × 10^-1^	1.26 × 10^-10^	CaM09	2.49 × 10^-1^	4.36 × 10^-38^
CaM31	DL					CaM42	5.94 × 10^-1^	7.46 × 10^-11^	CaM20	2.49 × 10^-1^	1.09 × 10^-38^
CaM34	DL					CaM58	6.14 × 10^-1^	4.58 × 10^-11^	CaM39	2.55 × 10^-1^	2.78 × 10^-39^
CaM35	DL					CaM17	6.40 × 10^-1^	2.93 × 10^-11^	CaM52	3.13 × 10^-1^	8.69 × 10^-40^
CaM39	DL					CaM30	6.40 × 10^-1^	1.87 × 10^-11^	CaM17	3.49 × 10^-1^	3.04 × 10^-40^
CaM43	DL					CaM08	6.52 × 10^-1^	1.22 × 10^-11^	CaM30	3.49 × 10^-1^	1.06 × 10^-40^
CaM55	DL					CaM09	6.52 × 10^-1^	7.97 × 10^-12^	CaM54	3.50 × 10^-1^	3.71 × 10^-41^
CaM57	DL					CaM20	6.52 × 10^-1^	5.20 × 10^-12^	CaM58	3.57 × 10^-1^	1.33 × 10^-41'^
CaM58	DL					CaM54	6.54 × 10^-1^	3.40 × 10^-12^	CaM06	3.71 × 10^-1^	4.92 × 10^-42^
CaM08	MM					CaM52	7.12 × 10^-1^	2.42 × 10^-12^	CaM42	3.71 × 10^-1^	1.83 × 10^-42^
CaM09	MM					CaM53	7.89 × 10^-1^	1.91 × 10^-12^	CaM53	4.49 × 10^-1^	8.21 × 10^-43^
CaM11	MM					CaM34	7.99 × 10^-1^	1.53 × 10^-12^	CaM34	5.00 × 10^-1^	4.10 × 10^-43^
CaM12	MM					CaM44	7.99 × 10^-1^	1.22 × 10^-12^	CaM44	5.00 × 10^-1^	2.05 × 10^-43^
CaM22	MM					CaM11	MM				
CaM23	MM					CaM13	MM				
CaM53	MM					CaM15	MM				
CaM54	MM					CaM23	MM				
Mean	6.09 × 10^-1^	--		2.67 × 10^-1^	--		5.19 × 10^-1^	--		1.68 × 10^-1^	--
SD (+)	1.57 × 10^-1^	--		2.10 × 10^-1^	--		1.30 × 10^-1^	--		1.52 × 10^-1^	--
SE (+)	3.36 × 10^-2^	--		4.47 × 10^-2^	--		1.99 × 10^-2^	--		2.32 × 10^-2^	--

**Note**: The markers are arranged as per their individual PI in the decreasing order; Cumulative power of discrimination was calculated using products of PIs of successive informative markers arranged in decreasing order as described by Waits et al. [56]. The PI was not estimated for DL and MM markers, as they were uninformative. DL: Duplicated loci; MM: Monomorphic markers.

#### Mappability of novel SSR markers

**Figure 2:**
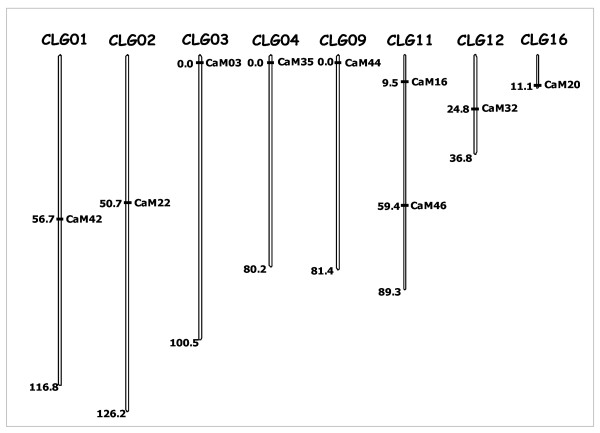
**Relative position of the nine new SSR markers (20% of the total tested) mapped on a robusta coffee map [12].** The reference map was generated using pseudo-testcross mapping population derived from a cross of 'CxR' (a commercial robusta hybrid) and Kagganahalla (a local selection from India). Note that the new mapped markers are distributed randomly across different linkage groups. The value at the base of each LG refers to its relative length in centiMorgans (cM).

The new SSR markers were tested for their mappability on robusta linkage map. In total, 9 of the 44 new markers (20.5%) were found to be polymorphic for the parents of the robusta pseudo-testcross mapping population *i.e*. CXR and Kagganahalla. The nine markers (CaM03, 16, 20, 22, 32, 35, 42, 44 and 46) could be mapped on the robusta linkage map developed by us [12]. Notably, seven of the markers (except CaM16 and CaM46) were mapped on independent LGs, which indicated the new markers to be randomly distributed on the robusta genome (Figure 2, Table 3).

#### Cross-species/-genera transferability and primer conservance

Cross species transferability of the new robusta derived SSR-markers was tested for 13 related *Coffea *and two *Psilanthus *species. In general, the markers resulted in robust cross-species amplifications with alleles of comparable sizes in the tested taxa (Table 4). Overall, an average transferability of ~92% was observed (Table 6, 7), which was higher for *Coffea *spp. (> 93%) than for the related *Psilanthus *spp. (~82%). Moreover, within different *Coffea *taxa, across its different botanical subsections, the transferability was comparable (> 91%). The data thus, indicated a very high marker conservance across the related coffee species, which was calculated to be ~91% over all the tested markers. Marker CaM54 exhibited lowest conservance of 23% (for *Coffea *species) and 27% (over all taxa), whereas 24 markers were found to be 100% conserved. The data also revealed the presence of some private alleles (PAs), which possibly could be species-specific. In total, 104 such alleles were found in *Coffea *(with a mean number of 8.7 PAs/species) and 35 in *Psilanthus *species (17.5 PAs/species), over all the 44 markers. These accounted for ~34% of amplified alleles in *Coffea *spp. and 45% of those amplified in *Psilanthus *spp.

**Table 6:** Conservation and transferability of the new SSR markers across related taxa of coffee

**Species**	*Coffea spp*.																	*Psilanthus *spp.			
			
	Erythrocoffea			Mozambicoffea					Pachycoffea							Melanocoffea		Paracoffea			
								
**SSR**	*C. arabica*	*C. congensis*	**Average C_*taxa*_**	*C. eugenioides*	*C. kapakata*	*C. racemosa*	*C. salavatrix*	**Average C_*taxa*_**	*C. excelsa*	*C. liberica*	*C. abeokuteae*	*C. dewevrei*	*C. arnoldiana*	*C. aruwemiensis*	**Average C_*taxa*_**	*C. stenophylla*	**Average C_*taxa*_(*Coffea*)**	*P. bengalensis*	*P. wightiana*	**Average C_*taxa*_(*Psilanthus*)**	**Average C_*taxa*_(for all coffees)**
CaM02	+	+	1.00	+	+	+	+	1.00	+	+	+	+	-	+	0.83	+	0.92	+	+	1.00	0.93
CaM03	+	+	1.00	-	+	+	+	0.75	+	+	+	+	+	+	1.00	+	0.92	-	+	0.50	0.87
CaM08	+	+	1.00	+	+	+	+	1.00	+	+	+	+	-	+	0.83	+	0.92	+	-	0.50	0.87
CaM09	+	+	1.00	+	-	+	+	0.75	+	+	+	+	-	+	0.83	+	0.85	-	-	0.00	0.73
CaM11	+	+	1.00	+	+	+	+	1.00	+	+	+	+	+	+	1.00	+	1.00	-	-	0.00	0.87
CaM18	+	+	1.00	+	+	+	+	1.00	+	+	+	+	-	+	0.83	-	0.85	+	+	1.00	0.87
CaM20	+	+	1.00	-	-	+	-	0.25	-	+	+	+	-	-	0.50	-	0.46	-	-	0.00	0.4
CaM23	+	+	1.00	+	+	+	+	1.00	+	+	+	+	+	-	0.83	+	0.92	+	+	1.00	0.93
CaM25	+	-	0.50	+	+	+	+	1.00	+	+	+	+	+	+	1.00	+	0.92	+	+	1.00	0.93
CaM31	+	+	1.00	+	+	+	+	1.00	+	+	+	+	+	+	1.00	+	1.00	-	+	0.50	0.93
CaM33	+	-	0.50	+	+	+	+	1.00	+	+	+	+	+	+	1.00	+	0.92	-	+	0.50	0.87
CaM36	+	+	1.00	+	+	+	+	1.00	+	+	+	+	+	-	0.83	+	0.92	+	-	0.50	0.87
CaM42	+	+	1.00	+	+	+	+	1.00	-	+	+	+	+	+	0.83	+	0.92	+	+	1.00	0.93
CaM45	+	+	1.00	-	+	+	+	0.75	+	-	+	+	+	+	0.83	+	0.85	+	+	1.00	0.87
CaM49	+	+	1.00	+	+	+	+	1.00	+	+	+	+	+	-	0.83	+	0.92	-	-	0.00	0.8
CaM53	+	-	0.50	-	+	+	+	0.75	+	-	+	+	+	-	0.67	+	0.69	+	+	1.00	0.73
CaM54	-	-	0.00	-	+	+	-	0.50	-	-	-	+	-	-	0.17	-	0.23	-	+	0.50	0.27
CaM55	+	+	1.00	+	+	+	+	1.00	+	+	+	+	+	+	0.83	-	0.85	+	-	0.50	0.8
CaM57	+	+	1.00	+	+	+	+	1.00	-	+	+	+	-	-	0.50	+	0.77	+	+	1.00	0.8
CaM58	+	+	1.00	+	+	+	+	1.00	+	+	+	+	+	+	1.00	+	1.00	-	+	0.50	0.93
24 SSRs other than listed above	+	+	1.00	+	+	+	+	1.00	+	+	+	+	+	+	1.00	+	1.00	+	+	1.00	1.00

***Average T*_*mark*_**	0.98	0.91	0.94	0.89	0.95	1.00	0.95	0.95	0.91	0.93	0.98	1.00	0.84	0.84	0.91	0.91	0.93	0.80	0.84	0.82	0.92 (***T*_*mark*-*taxa*_**)/0.91 (***C*_*taxa*-*mark*_**)

+/-: Indicates 'amplification'/'No amplification' and are given a weightage of 1 and 0 for transferability/conservence calculations respectively; T_*mark*_: Marker transferability over all the taxa; C_*taxa*_: Marker conservance over all the taxa; T_*mark*-*taxa*_: Marker transferability of all the markers over all the taxa; C_*taxa*-*mark*_: Primer conservance across all the taxa over all the markers.

**Table 7:** Plant materials used for validation and testing inter-specific/inter-generic transferability of new SSR markers

S.N.	Name of genotype	Pedigree/source
***I. Elite coffee genotypes used for genetic diversity in the cultivated genepool***		

1	Taferikela	*C. arabica*; Pureline from Ethiopian collections
2	HdeT	*C. arabica*; Amphidiploid coffee, a natural hybrid from *C. arabica *and *C. canephora*
3	S2790	*C. arabica*; HdeT × Tafarikela, selection
4	S2792	*C. arabica*; Tafarikela × HdeT, selection
5	S10	*C. arabica*; Double Cross Hybrid; Caturra with Cioccie and S.795 (both arabica)
6	S11	*C. arabica*; Amphidiploid, *C. liberica *× *C. eugenioides*
7	BM	*C. arabica*; Blue Mountain Pure line
8	Agaro-Sln4	*C. arabica*; Pureline from Ethiopian collections
9	Kagganahalla	*C. canephora*; Selection
10	BR9	*C. canephora*; Selection
11	BR11	*C. canephora*; Selection
12	CXR	*C. canephora*; Hybrid of *C. congenis *× *C. canephora*
13	L1Valley	*C. canephora*; Selection
14	S3329	*C. canephora*; Selection
15	S3334	*C. canephora*; Selection
16	Sln27	*C. canephora*; Pure line

***II. Parents and mapping population used for testing utility in mapping analysis***		

**Parents**: CXR (12) and Kagganahalla (9); **Mapping population**: 175 segregating progenies		

***III. Species of Coffea and Psilanthus (related taxa of cultivated coffee) used for transferability studies***		

a	*C. congensis*	Erythrocoffea (W. & C. Africa)
b	*C. excelsa*	Pachycoffea (Srilanka)
c	*C. liberica*	Pachycoffea (W. & C. Africa)
d	*C. abeokuteae*	Pachycoffea (Srilanka)
e	*C. dewevrei*	Pachycoffea (USDA)
f	*C. arnoldiana*	Pachycoffea (SanMarino)
g	*C. aruwemiensis*	Pachycoffea (SanMarino)
h	*C. eugenioides*	Mozambicoffea (C. Africa)
i	*C. racemosa*	Mozambicoffea (E. Africa)
j	*C. salvatrix*	Mozambicoffea (E. Africa)
k	*C. kapakata*	Mozambicoffea (C. Africa)
l	*C. stenophylla*	Melanocoffea (W. Africa)
m	*P. wightiana*	Paracoffea (India)
n	*P. bengalenis*	Paracoffea (India)

### Generic affinities within/between cultivated and wild coffee germplasm

The diploid microsatellite data were examined for their potential in genetic diversity studies by studying the variation and interrelationship between the cultivated as well as wild genepool. The average genetic distance values (calculated using the SSR allelic data) were found to be 0.26 (SD: ± 0.06; SE: ± 0.01), 0.43 (SD: ± 0.06; SE: ± 0.01) and 0.51 (SD: ± 0.17; SE: ± 0.02) for the tested arabicas, robustas and over both the sets, respectively. Similar estimates calculated for different *Coffea *and *Psilanthus *species were: 0.57 (SD: ± 0.12; SE: ± 0.04) for Erythrocoffea (diploid + tetraploid), 0.54 (SD: ± 0.07; SE: ± 0.05) for Erythrocoffea (diploids), 0.58 (SD: ± 0.05; SE: ± 0.02) for Mozambicoffea, 0.63 (SD: ± 0.09; SE: ± 0.02) for Pachycoffea, 0.65 (only two species, thus no SD) for Paracoffea, and 0.72 (SD: ± 0.10; SE: ± 0.01) over all the compared species.

**Figure 3:**
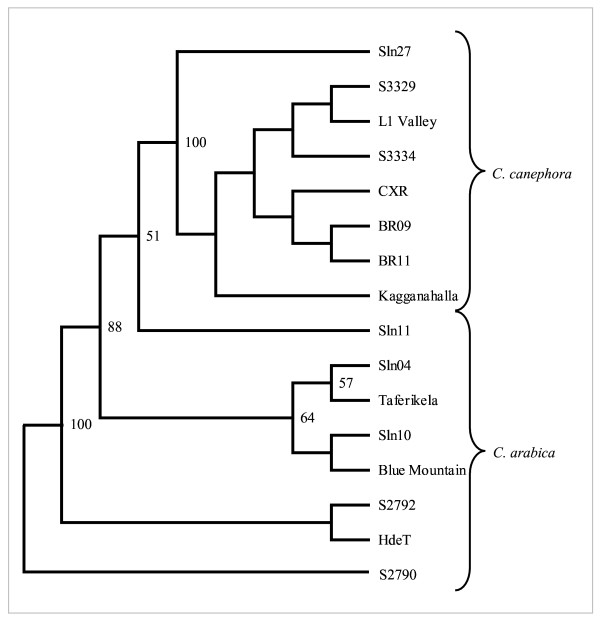
NJ tree showing relationship within and between arabica and robusta germplasm based on the allelic diversity generated using the new SSR markers.

**Figure 4:**
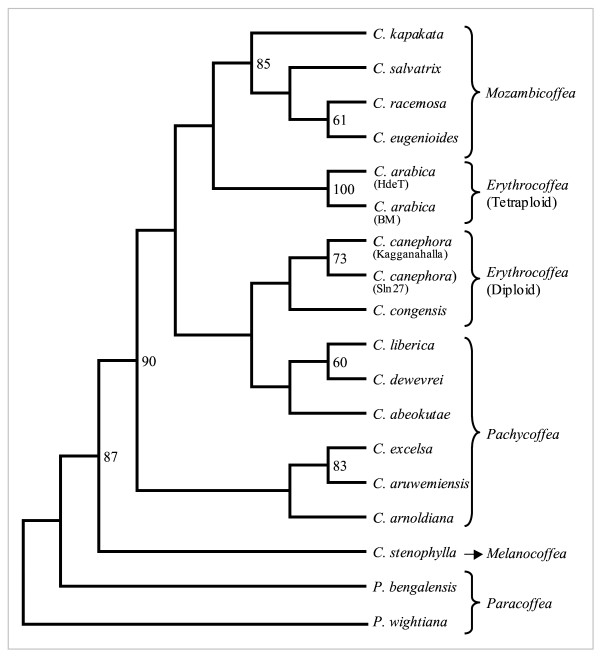
NJ tree showing relationship between 14 *Coffea *and two *Psilanthus *taxa based on the allelic diversity generated using the new SSR markers.

The NJ phenetic tree generated using the genetic distance estimates for eight genotypes each from arabica and robusta clearly resolved the tested germplasm in two distinct clusters, one representing all the tetraploid arabicas, while the other comprised all the diploid robustagenotypes (Figure 3) with significant branch support. The selections from pure arabicas formed a single cluster within arabicas, whereas selections from hybrids formed different group. HdeT was found closest to S2790 and S2792, whereas Sln11 was found to be the most distant entry in arabicas. Similarly, a clustering analysis of 14 related species (12 *Coffea *and two *Psilanthus *spp.; Figure 4) along with two genotypes each from *C. arabica *and *C. canephora *formed coherent clusters of diploid Erythrocoffeas (*C. canephora*, *C. congensis*), tetraploid Erythrocoffea (*C. arabica*), Mozambicoffea (*C. racemosa*, *C. eugenioides*, *C. salvatrix*, *C. kapakata*), and Pachycoffea (*C. liberica*, *C. dewevrei*, *C. abeokutae *as one cluster and *C. excelsa*, *C. arnoldiana*, *C. aruwemiensis *as other cluster). A single entry for Melanocoffea represented by *C. stenophylla *was the most divergent among the *Coffea *species and showed proximity with entries from Paracoffea section (*Psilanthus *spp.).

## Discussion

### Distribution and abundance of detected SSR motifs

The coffee-specific SSR markers described in this study were developed using the conventional approach of construction/screening of a partial small-insert genomic library. The success rate of any microsatellite development effort is indicated by the proportion of SSR-containing clones in the library followed by number of detected SSRs, qualities of SSR motifs and also by the quality of flanking regions. In the present study, 76 good quality SSR-positive clones containing a total of 116 SSRs were obtained from which 44 SSR markers were developed (Table 1, 3). The results, thus, suggested a success rate of 0.48% in the identification of potential target SSR-positive clones, and 0.28% in overall marker development. In a representative study to assess success of conventional library screening approach for microsat marker development in 16 different plant genera, it was found that the proportion of SSR-positive clones varied significantly (0.059% to 5.8% with an average of 2.5%) from species to species [14]. The observed SSR detection efficiency of the approach in this study was comparable with earlier reports in *Acasia *(0.32%, [15]) and peanut (0.43%, [16]), but was higher than rice (0.22%, [17]), potato, (0.06 to 0.15%, [18]) and wheat (0.11% [19]), and less than white spruce (0.62%, [20]).

The estimates derived from this study revealed that the relative distribution of different SSRs in robusta coffee genome is relatively poor in overall SSR abundance (1/160 Kb for targeted SSRs, and 1/15 kb including the non-targeted SSRs; Table 2) compared to various other plant species such as *Arabidopsis*, rice, barley (1 every 6–8 Kb) [21] and mulberry (our unpublished data). Nevertheless, the relative frequency, repeat lengths, and distribution pattern of different types of genomic SSRs in coffee genome (Table 2) were comparable to those reported in a number of plant species like apple [22], avacado [23], birch [24], peach [25], *Acasia *[15] and tomato [26]. In specific, AG was detected in higher proportion (almost 2 times) than AC; AG repeat cores were, in general, found to be longer than any other SSR type. Repeat cores of TNRs were, in general, smaller than DNRs, and AT (the non-targeted SSR) was found to be the most abundant in comparison to any other DNR or TNR. In comparison, the AT-rich TNRs in the coffee genome were found to be relatively less abundant than seen in most plant species [16,27,28], but comparable to some of the tree species like avacado (ACC > AGG > AAG, [23]) and peach (abundant in AGG, [25]). A species specific-pattern of TNR abundance has also been demonstrated in closely related species like rice and wheat that belong to the same family but differ significantly in their genomic TNR content [29-31]. Some of the variation seen in the SSR estimates (relative frequency, distribution and abundance) as discussed above across different studies including the present one on coffee, can be ascribed to the differences in criteria used for SSR search *viz*., minimum length of repeat-core, the size of the genomic library screened, screening stringency, oligos used for screening and SSR mining tools, notwithstanding the innate differences in genomic organization of SSRs in different species.

A comparison of the relative abundance/distribution of genomic SSRs with that of genic-SSRs developed from coffee transcriptome earlier by us [11], revealed two striking differences *viz*., an apparent higher abundance of SSRs in the transcriptome (1/2.16 Kb) and a near reverse pattern of TNR abundance/relative distribution in two types of SSRs. Importantly, the two most abundant TNRs (AAG, ACT) in the genic-SSRs were least abundant or not-detected in the genomic SSRs. The observation would suggest interesting possibilities of differential distribution/organization of TNRs as well as restriction sites for the enzymes used for library construction across gene-rich and gene-deficient regions of the coffee genome. However, such possibilities can only be addressed by further detailed genomic studies in times to come.

### Development of new SSR markers

In coffee, to the best of our knowledge till date only ca. 180 genomic SSRs have been described in literature [4-11] warranting continuous efforts to develop additional new markers to expand the existing repertoire for their efficient deployment in genetic studies in coffee. In this study 63% of the detected SSRs were found useful for primer design/marker conversion, a much higher success rate compared to that reported for apple (30% [22]), cassava (37.7% [32]), *Elymus caninus *(11.1% [33]), oat 25.2% [34] and potato (26.9% [18]). The two main sequence attributes that rendered 36 identified SSRs unsuitable for primer design were found to be: a shorter repeat core, and a low-complexity flanking region (AT/GC-rich and/or regions prone to secondary structure formation) unsuitable for primer modeling. Interestingly, in the present study, not even a single failure was due to the location of SSR-core towards the end of clone sequence, which is reported to be one major limiting factor in many earlier studies in cassava, tomato, oat and fir [26,32,34,35]. The higher success rate and less number of limiting factors in primer-designing observed in this study are expected to be due to the better suitability of the restriction enzymes, as well as, the relatively longer genomic fragments (0.5 to 1.5 kb) used for the genomic library construction. Importance of size of the genomic fragments used for construction of genomic library/SSR-marker development has also been shown earlier in groundnut [16].

The proportion of designed primers successfully producing amplification products gives a primer-to-marker conversion ratio and indicates the ultimate success of the library construction effort. In this study, of the 58 primer pairs designed, 44 could be validated as efficient SSR-markers (see Tables 3, 4, and the discussion in the following sections) thus resulting in ~75.8% primer-to-marker conversion ratio, broadly comparable to many earlier conventional genomic library-based studies *viz*., cucurbits [36], *Elymus *[33], peanut [16], tomato, [26] and rice [17]. One of the lowest primer-to-marker convertibility reported for Douglas fir (4.1%) was suggested to be due to the complexity unique to the conifer genomes [35,37-39]. Further, a survey of the literature suggests, in general, a higher conversion ratios for small genomes like apple, peach, and a negative correlation between the genome size and the amplification efficiency of SSR primers due to mechanistic reasons [40].

Two of the 44 new SSR markers described here (CaM49, 55) were based on MNR repeats. In general, these markers warranted much more critical appraisal for ascertaining their individual alleles/sizing that in many cases were not easily distinguishable from the similar sized confounding stutter amplicons (data not shown). Therefore, it may be prudent to avoid use of such MNR-based markers despite these being informative, unless no other markers are available.

### Utility of new SSRs as genetic markers

Till date, there are a few studies describing development of coffee-specific SSR markers [4-11]; however, only a few of these provide data for the utility of new SSRs in genetic studies [8,11]. Therefore, one major aim of the present study was to test the potential of the new markers reported here for their use in studies related to genetic diversity in cultivated coffee germplasm, linkage mapping, constructing reference panels/bar codes for individualization of genotypes, cross-species transferability, and taxonomic relationship in related taxa.

#### Germplasm characterization

##### Level of allelic polymorphism and genetic diversity

Various genetic parameters *viz*., allelic diversity, PIC, H_o_, H_e_, Kurtosis/skewness, HWE, LD, calculated for all the new SSRs amply demonstrated their utility as genetic markers (see results, Table 4). In general, the markers revealed low to moderate allelic/genetic diversity which was comparable and in some cases more than that reported for the earlier described coffee genomic SSRs [6,8], and as expected, invariably higher than the genic-SSRs [10,11,41]. The total number of alleles amplified by different markers in the tested arabicas and robustas was almost similar; however, the markers were found significantly more informative with higher PIC values for robustas. In addition, it was important to note that 13 of the tested markers amplified two distinct but similar sized alleles across all the tested arabicas suggesting these to be the result of duplicated fixed loci in the arabica genome. The above observations are likely considering the reproductive behavior, genome evolution and domestication process of two types of coffee. The robustas are expected to be genetically more diverse (leading to higher PIC for tested markers) due to their out-crossing behavior in contrast to arabicas that are self-compatible and also known to suffer from narrow genetic base resulting from the genetic bottleneck during domestication process [8,11]. Similarly, the duplicate loci in arabica genome are plausible as it is an allotetraploid resulted from hybridization of two homeologous diploid genomes (*C. eugenioides *and *C. canephora*) followed by diploidization and stabilization [42].

Different genetic parameters/tests such as H_o_, H_e_, LD, HWE are important indicators of origin, evolution and distribution of diversity in the available genepool. The heterozygosity measures (H_o_, H_e_) for the new SSR markers indicated significant heterozygote decay (deficiency) in the tested germplasm. Kurtosis/skewness parameters indicated that the allelic diversity for the new SSRs does not follow normal distribution. Similarly, the HWE and LD analysis of the polymorphic markers (P*m*s) revealed only about 2/3^rd ^of the markers (63 – 65 %) in HW equilibrium and about 25–29 % markers showing significant LD in the analyzed arabicas and robustas. These results are in agreement with our earlier observations with genomic as well as genic-SSRs [6,10,11], and indeed reflective of the genetic composition and mating behavior of the tested materials. Overall, these studies indicated that the tested robusta germplasm comprised allogamous, relatively unrelated genotypes (selections and one hybrid), while autogamous arabicas comprised mostly of hybrid varieties/selections with overlapping/shared pedigrees. The results thus suggest the suitability of the new markers for reliably ascertaining genetic diversity in the coffee genepool.

##### Discriminatory power of new SSR markers

Individualization of plant germplasm resources has become important in the present day scenario for their proper management and utilization, as well as IPR protection which can be achieved by DNA typing techniques involving use of highly polymorphic markers like SSRs. Germplasm characterization using such typing approaches remains a costly proposition, especially if the target species like coffee that has very limited diversity in its available genepool. To circumvent these problems and increase the utility of such efforts, it has been proposed to build reference DNA polymorphism data resources/panels for coffee germplasm using robust markers like SSRs and common experimental guidelines [12,43]. Such reference resource can then readily be used for coffee genotype individualization, germplasm selection for breeding/improvement, and germplasm exchange in international collaborations [12,43]. In this context, it becomes important to ascertain the PI estimates (that provide very informative indicators of the discrimination potential) of the SSR markers, before deployment in germplasm characterization studies. In general, the PI estimates for the new markers ranged from low to moderate when considered individually, but were highly informative for genotype discrimination when tested together (cumulative PI).

Moreover, the estimates were found to be reflective of the diversity status in the test germplasm, and accordingly were significantly different (lower) for arabicas than the robustas (Table 5). The analysis in general indicated the need for use of 3–4 times more markers to achieve the comparable level of discrimination in the two coffee genepools. Moreover, the data suggested that from practical point of view it might be prudent to calculate the sib-based PI (a more conservative estimate of discrimination) for deciding the number of markers that can provide sufficient variability for individualization of the test germplasm. This is expected as the sib-based PI discounts the possible similarities/relatedness in the target germplasm arising due to overlapping pedigrees/common parentage.

#### Mappability of the new SSR markers

One of the major potential utilities of DNA markers is their use as robust genomic landmarks on the linkage groups that can subsequently be tagged to the gene(s) controlling important traits of interest providing possibilities of MAS-based breeding. This requires generation of reasonably dense linkage maps populated with large number of revisitable DNA markers for which the SSRs remain the most desired ones. Till date, very few SSRs are mapped on the robusta linkage map [7,44] warranting extensive efforts to generate more SSR markers usable for linkage analysis. In this regard, we tested the suitability of the new markers for linkage mapping using a pseudo-testcross mapping population of robusta coffee. Significantly, 20.5% of the markers were found to be polymorphic for the parents of the mapping population, and all of these could be successfully mapped (Figure 2). The mapped markers were distributed on different linkage groups, and some of these mapped towards the ends of the LGs as has been seen in the earlier studies [44]. The data, thus, strongly demonstrate that the new markers can be efficiently used for genetic linkage studies in coffee.

#### Cross-species/-generic transferability

The low-moderate level of diversity exhibited by the new markers in the cultivated coffee genepool, is more than compensated by their high potential for cross-species transferability. All the markers revealed robust cross-species/-generic amplifications with alleles of comparable sizes when tested for 13 *Coffea *and two *Psilanthus *taxa (Table 7). The data revealed that the markers described here show much better taxa transferability than the earlier published genomic SSR markers [6,9,10], but relatively less than the genic SSR markers reported by us [10,11]. More importantly, the markers showed comparable transferability across related species of *Coffea *as well as 2 species of the related genus *Psilanthus*. This is significant as successful cross-species amplification is generally restricted to related species within a genus and reduces when tested for different genera [11,45]. Further, it was interesting to note that all the new SSRs that were monomorphic/uninformative for the tested arabica and robusta germplasm, exhibited considerable polymorphism across the tested related taxa. The only exception was the marker CaM54 that showed a very low conservance even across the *Coffea *spp. Thus, the new SSR markers described here strengthen the possibility of their use as Conserved Orthologous Sets (COS) for genetic characterization of different related wild coffee taxa, and also for coffee taxonomic/synteny studies.

#### Diversity analysis and genetic relatedness within/between Coffea and Psilanthus species

The genomic SSRs described in this study, despite revealing low level of polymorphism, were able to group all the 16 genotypes belonging to two cultivated germplasms in phenetic clustering that were indicative of species relationship and confirming their known pedigrees (Figure 3). For example, the analysis confirmed the related origin of S2790 and S2792, which are two-way hybrids between HdeT and Taferikela.

Similarly, the analysis of 20 representative samples belonging to 14 *Coffea *and two *Psilanthus *species, revealed generic affinities that were in general agreement with their known taxonomic relationships, based on their geographical distribution as well as Chevalier's botanical classification [46] (Figure 4). Accordingly, the phenetic tree based on the new markers data very clearly grouped the analyzed related coffee species as per their respective botanical sub-sections (see results). Importantly, the analysis distinctly separated the two Paracoffea species (*P. bengalensis *and *P. wightiana*) from all the other *Coffea *spp. These results are similar to the earlier published studies undertaken to ascertain species relationships using SSRs [8,9,11], as well as other marker approaches [47-49]. A close relationship of *C. kapakata *to the Mozambicoffea taxa, and status of the only Melanocoffea taxon *C. stenophylla *as seen here was also indicated earlier in the EST-SSR and ISSR-based studies [11,47]. These results, thus, demonstrate that the new SSR markers developed in the present study can be highly informative in exploring the taxonomic relationship of coffee species complex.

## Conclusion

In summary, the present study describes 44 new microsatellite markers developed using the conventional approach of construction/screening of partial small-insert genomic library. The approach was found to be successful but difficult and experiment-intensive with low success rate of ~0.48%. Analysis of the identified SSR-positive genomic clones provided insights into the relative abundance, and distribution pattern of different SSR motifs in the coffee genome that was found to be relatively poor in its SSR abundance compared to many other plant genomes. Overall, the DNRs were much more abundant than TNRs, and among different types of SSR motifs, AT was the most abundant followed by AG, AC, and ACG. The TNR CCG, was the least abundant. More than 50% of the identified SSRs could be converted to usable markers resulting in a high primer-to-marker conversion ratio. All the 44 markers were found to be polymorphic in the tested coffee/related germplasm and their utility as efficient genetic markers could be demonstrated for diversity analysis, germplasm individualization, linkage mapping, cross-species transferability and taxonomic studies. This study has thus enriched the available small repertoire of coffee SSR markers by 44 new SSRs, which are not only useful for cultivated coffee but are also expected to be equally useful for genetic studies involving related species that constitute the important secondary genepool for improvement of coffee.

## Methods

### Plant material and DNA extraction

In this study sixteen elite genotypes belonging to *C. arabica *and *C. canephora *were used along with 14 related wild species belonging to *Coffea *and *Psilanthus *genera (Table 7). The leaf samples for each of them were collected from germplasm bank maintained at Central Coffee Research Institute, Balehonnur, Karnataka, India and DNA was isolated following the method described by Aggarwal et al. [50].

### Construction of genomic library and isolation of SSR containing sequences

A partial small-insert genomic library was constructed using standard procedures [51] from total cellular DNA isolated from an elite robusta genotype, Sln-274. Approximately 10 μg of genomic DNA was digested with *Rsa *I and *Hae *III (NEB) restriction endonucleases (NEB, USA) and fractionated in 1% agarose gel. Genomic fragments of 500 to 1500 bp were gel-excised, purified using the GFX column (Amersham), ligated to pMOS Blunt-ended plasmid vector (Amersham) using T4 DNA-ligase, and finally the ligated genomic inserts were cloned in *Escherichia coli *DH10B host cells by electroporation. The transformed cells were grown overnight and recombinant white colonies were individually picked up and maintained in forty one 384-well microtiter culture plates, and replicated onto Hybond-N^+ ^nylon membranes (Amersham Biosciences, USA) to obtain high-density hybridization filters for screening. All the 15,744 arrayed recombinant clones were Southern hybridized to γ-^32^P-labeled two oligo pools (each comprising different synthetic oligonucleotides in equimolor concentration), *viz *Pool-I: (CA)_15_, (GA)_15_, (CAA)_10_, (CAT)_10_, (ACT)_10_, (GATA)_10_, (AGA)_10_, (CATA)_10_; and Pool-II: (CTG)_10_, (GAC)_10_, (AGG)_10_, (GGT)_10_, (GCC)_10_, (GC)_15_. Hybridized clones were selectively picked up and individually processed for plasmid isolation following the standard alkaline lysis method [51]. The genomic inserts were then amplified and sequenced using M13 universal primers for both the strands on 3700 DNA Analyzer using BigDye™ chemistry as per the manufacturer's details (Applied Biosystems, USA). The sequences were aligned and edited using Autoassembler (Applied Biosystems, USA) and finally saved in FASTA format.

### Marker Development

The identification and localization of microsatellites in the sequenced clones was performed using microsatellite search module MISA (for more information please see Availability & requirements section below) followed by visual assessment. Criteria for SSR search by the *MISA *were repeat stretches having a minimum of: 12 repeat units for MNRs, six repeat units in case of DNRs and five repeat units for HO-NRs. The microsatellites were classified considering the complementarities of the repeat motifs, e.g., AG, GA, TC and CT were considered as a single category. Primer pairs were designed for the SSR containing sequences with minimum of seven DNRs, and/or five repeats for all other SSRs using GENETOOL Lite version 1.0 (for more information please see Availability & requirements section below). The primers were commercially synthesized (Bioserve, India – for more information please see Availability & requirements section below) with forward primers having the fluorescent label FAM or HEX. The details of these new markers *viz*., locus designation, primer sequences, repeat motifs, allele attributes, PIC estimates and Genbank accession numbers, are summarized in Table 3, 4. The primer pairs were standardized and PCR was performed as described earlier [10,11]. The amplified products were run on capillary-based 3730 DNA Analyzer (Applied Biosystems) and the products were precisely sized for major, comparable and conspicuous peaks using GeneMapper 3.7 (Applied Biosystems), using default parameters.

### Statistical and genetic analysis

The allelic data for eight genotypes each for arabicas and robustas were used to calculate different statistical and genetic parameters. The statistical attributes like mean, skewedness, kurtosis, t-test *etc*. were calculated using Microsoft Excel function utilities. Observed heterozygosity (H_o_) was calculated as fraction of heterozygous genotypes over total number of genotyped plants. Expected heterozygosity (H_e_) was calculated according to the following formula [52]:

H_e _= (n/n-1)(1-Σpi^2^).

PIC values were calculated according to Botstein et al. [53] as follows:

1-Σpi^2^-ΣΣ2pi^2^pj^2^,

where,

n = the total number of alleles detected for a microsatellite marker,

Pi = the frequency of the i^th ^allele, and

pj = the frequency of the (i+1)^th ^allele in the set of analyzed genotypes.

The bi-allelic polymorphic data were also tested for Hardy-Weinberg equilibrium (HWE) using Fisher's exact test and Markov chain algorithm with forecasted chain length of 10,000,000 and 100,000 dememorization steps and linkage dis-equilibrium (LD) test was performed using 1,000 permutations. For arabicas, the markers that showed invariable presence of 'double alleles' across the tested germplasm were considered as independent amplifications from duplicated loci present in two distinct copies and were excluded from the analysis for the allelic attributes described above. The H_o_, H_e_, estimates and HW and LD tests were done using the program Arlequin ver 3.1 [52], and the probability of identity (PI) estimates were calculated using the program Gimlet ver 1.3.2 [54]. Private alleles (PAs) were determined using the software Convert ver. 1.3.1 [55] over all the 30 genotypes. The discriminatory power of each microsatellite locus was calculated by estimating sib-based and unbiased corrected PI estimates and cumulative power of discrimination was calculated as products of PIs of successive informative markers arranged in decreasing order as described by Waits et al. [56]. Cross-taxa transferability (T_*mark*_) was calculated as proportion of primers showing successful amplification *vis-à-vis *all the tested primers, whereas primer conservance (C_*taxa*_) was calculated as proportion of the species displaying successful amplification *vis-à-vis *all the tested markers.

The average genomic distance estimates between the detected SSR motifs were obtained by considering random sampling of the genome. Thus, for targeted SSRs, size of the sampled genome was considered equal to the total size of screened library, whereas for the non-targeted SSRs, the size of genome actually sequenced was used to get the estimates, considering the haploid coffee genome equivalent to 809 Mb [13]. Initially, the number of different DNRs and TNRs present in the robusta genome were estimated from the screened genome for targeted SSRs and the sequenced genome for non-targeted SSR *i.e*. AT-DNR. These were further used to estimate distribution of different SSRs in terms of SSR per Mb of the genome, and also as spacing between two such consecutive SSR repeats in robusta genome.

The linkage map was constructed using JoinMap ver 3.0, at LOD 5.0 and other default parameters as per the software instructions. The segregating allelic data was scored for the tested microsatellites as per the models specified in JoinMap for co-dominant marker-segregation in a pseudo-testcross population. The segregation data obtained in this study was used along with the mapping data available for the reference robusta population in the lab (unpublished).

### Genetic Diversity Analysis

The SSR data from P*m*s were used to ascertain the generic relationships/affinities between the tested germplasm (cultivated genotypes/related species) using cluster analysis based on genetic distance values. Initially 100 bootstrap distance matrices were generated using bi-allelic microsatellite data analysis tool, MicroSatellite Analyzer (MSA) [57] and Nei's genetic distance measure [58]. From these distance data, neighbour joining (NJ) trees were generated for each matrix separately using Phylip ver 3.6 [59] by 'neighbor' command, which was followed by generation of consensus trees, one each for the cultivated germplasm and inter-species relationships.

## List of abbreviations

DNRs: Di-Nucleotide Repeats; C_*taxa*_: Conservation of markers across the tested taxa; COS: Conserved Orthologous Sets; He: Expected heterozygosity; Ho: Observed heterozygosity; HO-NRs: Higher Order Nucleotide Repeats; HNRs: Hexa-Nucleotide Repeats; HWE: Hardy-Weinberg Equilibrium; Kb: Kilobases; LD: Linkage Disequilibrium; SSR: Simple Sequence Repeat; Mb: Megabases; MNRs: Mono-Nucleotide Repeats; MSA: MicroSatellite Analyzer; N_A_: Number of Alleles NJ: Neighbour Joining; PAs: Private Alleles; PIC: Polymorophism Information Content; PI: Probability of Identity; P*m*s: Polymorphic Markers; TNRs: Tri-Nucleotide Repeats; T_*mark*_: Transferability of markers across all the studied taxa; TtNRs: Tetra-Nucleotide Repeats.

## Authors' contributions

PSH constructed, screened the library and sequenced positive clones; developed and standardized majority of the markers; validated and analyzed the data; drafted the manuscript. PR, AL and AV standardized and validated some of the markers and helped in analysis. RKA conceptualized, planned, supervised, finalized, approved and communicated the final manuscript.

## Availability & requirements

1 MIcroSAtellite: 

2 GENETOOL Lite version 1.0: 

3 Bioserve, India: 
